# Dynamic versus static medial patellofemoral ligament reconstruction technique in the treatment of recurrent patellar dislocation: a randomized clinical trial protocol

**DOI:** 10.1186/s13018-022-03158-6

**Published:** 2022-07-10

**Authors:** Anna Bartsch, Corina Nüesch, Bertram Rieger, Annegret Mündermann, Christian Egloff

**Affiliations:** 1 Department of Orthopedics and Traumatology, University Hospital Basel, Spitalstrasse 21, 4031 Basel, Switzerland; 2 Department of Biomedical Engineering, University Hospital Basel, Basel, Switzerland; 3 Department of Clinical Research, University of Basel, Basel, Switzerland; 4 Department of Spine Surgery, University Hospital Basel, Basel, Switzerland; 5 Orthopedic Surgery and Sportsmedicine, ALTIUS Swiss Sportmed Center, Rheinfelden, Switzerland

**Keywords:** Medial patellofemoral ligament, MPFL, MPFL reconstruction, Patella instability, Knee biomechanics, Patellofemoral joint, Surface EMG

## Abstract

**Background:**

The redislocation rate of conservatively treated patella instability is high. One of the leading surgical strategies is medial patellofemoral ligament reconstruction. Over-tensioning is one of the most challenging complications in static medial patellofemoral ligament reconstruction as the graft used for reconstruction is isometric and the anatomical MPFL is a mostly dynamic structure. As an alternative to established static reconstruction techniques, dynamic graft techniques have been introduced for stabilizing the patella with the aim of providing a more physiological reconstruction of the medial patellofemoral ligament. To date, data on clinical outcomes are scarce and on biomechanical outcomes of the dynamic MPFL reconstruction are lacking. Here, we present the protocol of a randomized clinical trial for comparing clinical and biomechanical outcomes of dynamic versus static medial patellofemoral ligament reconstruction.

**Methods:**

This study is a prospective, single blinded, randomized, multicenter, multimodal (clinical and biomechanical) clinical trial. Patients with recurrent patella dislocation requiring isolated MPFL reconstruction will be recruited and randomized to the dynamic or static reconstruction technique. Participants will be followed up for 2 years with a total of five follow-ups. Preoperative magnetic resonance imaging, upright radiographs, surgical reports and patient records will be evaluated, and clinical and functional outcomes will be measured. Patient-reported knee function and anterior knee pain as assessed with the Kujala score will serve as primary outcome. For biomechanical outcome, pre- and postoperative evaluations will be performed to assess isokinetic muscle strength, gait asymmetry, joint kinematics and kinetics, and timing of muscle activity.

**Discussion:**

The results of the study will clarify whether the reported surgery success for patella stabilization via dynamic MPFL reconstruction is due to muscle contraction or to the passive tenodesis effect combined with clinical outcome measures. With this study, we will provide much needed information on knee biomechanics after dynamic versus static MPFL reconstruction to provide evidence to support orthopedic surgeons in evidence-based decision-making in their quest for surgical techniques most favorable for their patients.

*Trial registration* The study protocol was registered at clinicaltrials.gov (NCT04849130). Registered 19 April 2021, https://clinicaltrials.gov/ct2/show/NCT04849130.

## Background

High joint reaction forces in the patellofemoral joint of up to several times body weight [1, 2] are reflected in patellar cartilage being the thickest in the body with a patellar cartilage thickness of up to 7.5mm [3]. Articular cartilage damage is common, with 44.6% cartilage lesions of the patellofemoral joint reported in knee arthroscopies [4]. The pathogenesis of these lesions may result from acute trauma or altered joint loading due to patella instability. After patella dislocation, the incidence of acute osteochondral or chondral injuries is up to 95% after initial patella dislocation [5]. Moreover, chronic cartilage damage has been described at 13-year follow up with patellofemoral osteoarthritis in 22% in patellar instability knees compared to 11% in contralateral healthy knees [6].

Misshaped structures such as a high riding patella (patella alta) [7] or trochlear dysplasia [8] are risk factors for patella instability. The medial patellofemoral ligament (MPFL) has been shown to be the main dynamic stabilizer of the patella against lateral translation [9], especially when the patella has not yet engaged in the trochlear groove (knee flexion < 30°) [10]. After initial patellar dislocation, the MPFL is injured in 94% of the cases [11]. Because the redislocation rate of the patella after conservative management reaches up to 67% [12, 13] most authors recommend surgical management of these cases, and a variety of surgical procedures has been developed [14].

**Fig. 1:**
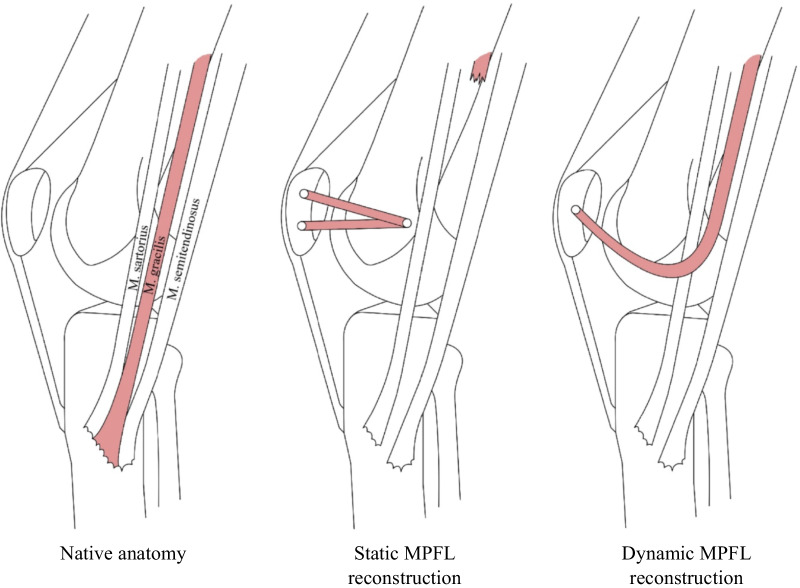
Native anatomy, static and dynamic MPFL reconstruction

The leading surgical strategy is to stabilize the patella through a reconstruction of the insufficient or disrupted soft tissue of the MPFL. This is necessary in recurrent patella dislocations and in particular cases of first patella dislocations such as patients with severe anatomical risk factors [15]. The anatomical risk factors should be corrected as well because these patients have an increased rate of revision surgeries, re-dislocations and persistent joint instability compared to those without anatomical risk factors [16]. Moreover, a prolonged time from dislocation to surgery appears to be associated with increased risk of re-dislocations [17]. To date, experts disagree regarding the treatment of complex patella instability [18]. In MPFL reconstruction, a graft (harvested autologous tendon or allograft) intended to stabilize the patella is placed using tunnels, screws, and/or anchors. While the use of interference screws versus suture anchors for isolated MPFL reconstruction has been shown to have similar clinical outcomes, the use of screws was associated with slightly higher complication rates [19]. One established procedure is the static MPFL reconstruction according to Schöttle et al. [20] (Fig. 1), who use the autologous gracilis tendon to stabilize the patella with a static reattachment to the femur. Despite good long-term outcomes of the static MPFL technique [16, 21–23] for up to 4 years, there remains a risk of malpositioning or over-tensioning the graft [24]. While too low graft tension leads to recurrent instability and failure, too high graft tension can lead to persistent knee pain and result in higher patellofemoral pressure, which may ultimately lead to patellofemoral joint degeneration and osteoarthritis [22, 25–27]. Over-tensioning is one of the most challenging complications—even in clinically adequately placed MPFL reconstructions—as the graft used for reconstruction is isometric, whereas the anatomical MPFL is a mostly dynamic structure [28] being tight in extension and early flexion and nearly isometric beyond 30° of flexion [24].

Several studies have addressed the challenges of correct isometric graft tensioning by evaluating the appropriate load and manner of optimal graft tension during surgery and ways of determining the optimal length and elasticity of the graft [24, 26]. As an alternative to static reconstructions, more elastic insertion techniques with soft tissue fixation only have been suggested [29–33] where the static graft is attached to soft tissue allowing small movements. The medial patellotibial ligament (MPTL) is considered as another restraint of patellar stability and often reconstructed as well in soft tissue fixation procedures [33, 34]. Becher et al. [29] have suggested that not only a dynamic insertion but also a dynamic rather than a static graft should be used for stabilizing the patella. They described a fully dynamic MPFL procedure by detaching and reinserting only the distal part of the gracilis muscle to the patella and leaving the proximal tendon attached to its muscle presumably facilitating dynamic adjustment of the patellar position by muscle contraction (Fig. 1). In a retrospective cohort study, Becher et al. [29] reported comparable mean outcomes of static and dynamic MPFL reconstruction are measured by Kujala, Tegner and Lysholm scores with large variability among groups. The advantage of this dynamic graft procedure is an improved physiological reconstruction of the MPFL as a mostly dynamic structure. Moreover, several surgical steps of the static MPFL reconstruction are avoided, and the dynamic MPFL procedure is therefore faster, simpler and more cost effective. While the advantages of shorter and more forgiving surgical technique in the Becher's procedure seem to make it preferable to other techniques, to date high-quality data on clinical and functional outcomes are lacking.

In this prospective, single blinded, randomized, multicenter clinical trial, we will compare clinical and biomechanical outcomes of static MPFL reconstruction according to Schöttle et al. [20] and dynamic MPFL reconstruction according to Becher et al. [29] up to 2 years after surgery.

## Methods/design

### Specific aims

The dynamic MPFL reconstruction is a muscle transfer procedure, and we assume that the function and timing of the gracilis muscle activity will change after dynamic MPFL reconstruction. We will compare longitudinal clinical and biomechanical outcomes (Table 1) between two major existing techniques: static reconstruction technique according to Schöttle et al. [20] and dynamic reconstruction technique according to Becher et al. [29].

**Table 1:** Procedures summary

Project period	Pre-hospitalization/Screening	Hospitalisation	Follow-up				
Assessment	1	2	3	4	5	6	7
Time	Day 0	Length of stay	6 weeks ± 2 weeks	12 weeks ± 3 weeks	6 months ± 4 weeks	12 months ± 4 weeks	24 months ± 4 weeks
Diagnostics (X-Ray, MRI as indicated)	x						
Informed Consent (Surgery and study)	x						
In-/Exclusion Criteria	x						
Pre-op. Physical examination	x	x (Day 0)					
Data to be provided by the treating clinician							
Intervention (CHOP Code)		x (Day X_1_)					
Complications		x (Day 0-X_2_)	x	x	x	x	x
Physical examination	x	x	x	x	x	x	x
Data to be extracted from routine records							
Demographics		x (Day 0)					
Medical history and medication		x (Day 0)					
Radiographic parameters	x						
Length of stay		x (Day 0)					
Complications		x (Day X_2_)					
Data to be collected by the study nurse in cooperation with the patient							
Kujala, Tegner, Lysholm score, Banff-score		x (Day 0)	x	x	x	x	x
Patient satisfaction		x (Day X_2_)	x	x	x	x	x
End of rehabilitation/end of physiotherapy			x	x	x	x	x
Data to be collected at the Functional Biomechanics Laboratory in cooperation with the patient							
Isokinetic muscle strength	x					x	
Gait analysis	x					x	

#### Specific Aim 1

Compare the clinical outcome of dynamic and static MPFL reconstruction.

#### Hypothesis 1

Patients with dynamic MPFL reconstruction will report equal or better patient reported outcomes compared to patients with static MPFL reconstruction, as assessed with the Kujala score [35] (primary outcome), Banff-II-score [36], IKDC-2000 [36] and the EQ-5D-5L [37]. Patients with dynamic MPFL reconstruction will have comparable redislocation rates. The few studies published to date [29, 38] show good or better clinical results with the dynamic MPFL reconstruction.

#### Specific Aim 2

Evaluate and compare the biomechanical outcomes of dynamic and static MPFL reconstruction.

#### Hypothesis 2

Patients with dynamic MPFL reconstruction will have less asymmetry in muscle strength and lower extremity gait kinematics and kinetics than patients with static MPFL reconstruction. To date, comparable biomechanical data of the dynamic MPFL reconstruction technique do not exist.

#### Specific Aim 3

Determine whether the reported success for patella stabilization in dynamic MPFL reconstruction is due to muscle contraction or to the passive tenodesis effect.

#### Hypothesis 3

The function of the gracilis muscle will change from a knee flexor with normal activity from pre-swing throughout the entire swing phase to the beginning of the loading response after the surgery to an isolated patella stabilizer, specifically depending on the knee flexion angle. We anticipate that muscle activity will shift more toward the loading response and the whole stance phase at knee angles < 30° of flexion. The capability and efficacy of such muscle transfer procedures have been described for other procedures such as rectus femoris transfer in children with cerebral palsy [39, 40]. The gracilis muscle reaches forces up to 300 N from 0–60° knee flexion [41] indicating that the gracilis muscle is adequate for patella stabilization.

### Study design

This study is a prospective single blinded randomized, multicenter, multimodal (clinical and biomechanical) clinical trial.

### Participants

We will examine 60 patients undergoing isolated MPFL reconstruction due to patella dislocation. The patients will be randomly assigned to the procedure (static reconstruction technique according to Schöttle et al. [20] or dynamic reconstruction technique according to Becher et al. [29]) using an a priori generated list of codes assigned in five blocks of six codes per center generated with a block randomization generator (www.sealedenvelope.com). Patients and the staff collecting questionnaires or biomechanical data will be blinded to the surgical procedure.

#### Inclusion criteria

Inclusion criteria are: isolated MPFL reconstruction due to patella instability in patients with closed growth plates; patella dislocation; Patella-Instability-Severity (PIS) score [42] ≤ 3 with concomitant flake fracture; PIS score ≤ 4 with clinical asymptomatic trochlea dysplasia (patella stability between 30 and 60° knee flexion); and no other clinically relevant static risk factors as patella alta or increased tibial tuberosity trochlear groove distance.

#### Exclusion criteria

Exclusion criteria are: combined procedures with trochleoplasty (trochlea dysplasia Type C or D according to Dejour et al. [43] with clinical instability between 30 and 60° of knee flexion); combined procedures with cartilage transplantation; high grade patellofemoral arthritis (Kellgren Lawrence [44] score ≥ 3); combined procedures with femoral or tibial osteotomy; clinically eminent valgus axis (> 15° valgus); femoral internal rotation > 20°; tibial external rotation > 40°; instability of the cruciate or collateral ligaments; known significant musculoskeletal disease or cognitive impairment; and patient age < 14 years.

#### Ethical considerations

The study protocol was approved by the regional ethics board (Ethics Committee Northwest Switzerland EKNZ 2020-02701) and registered at clinicaltrials.gov (NCT04849130). Written informed consent will be obtained from all participants prior to participation.

## Clinical trial protocol

**Fig. 2:**
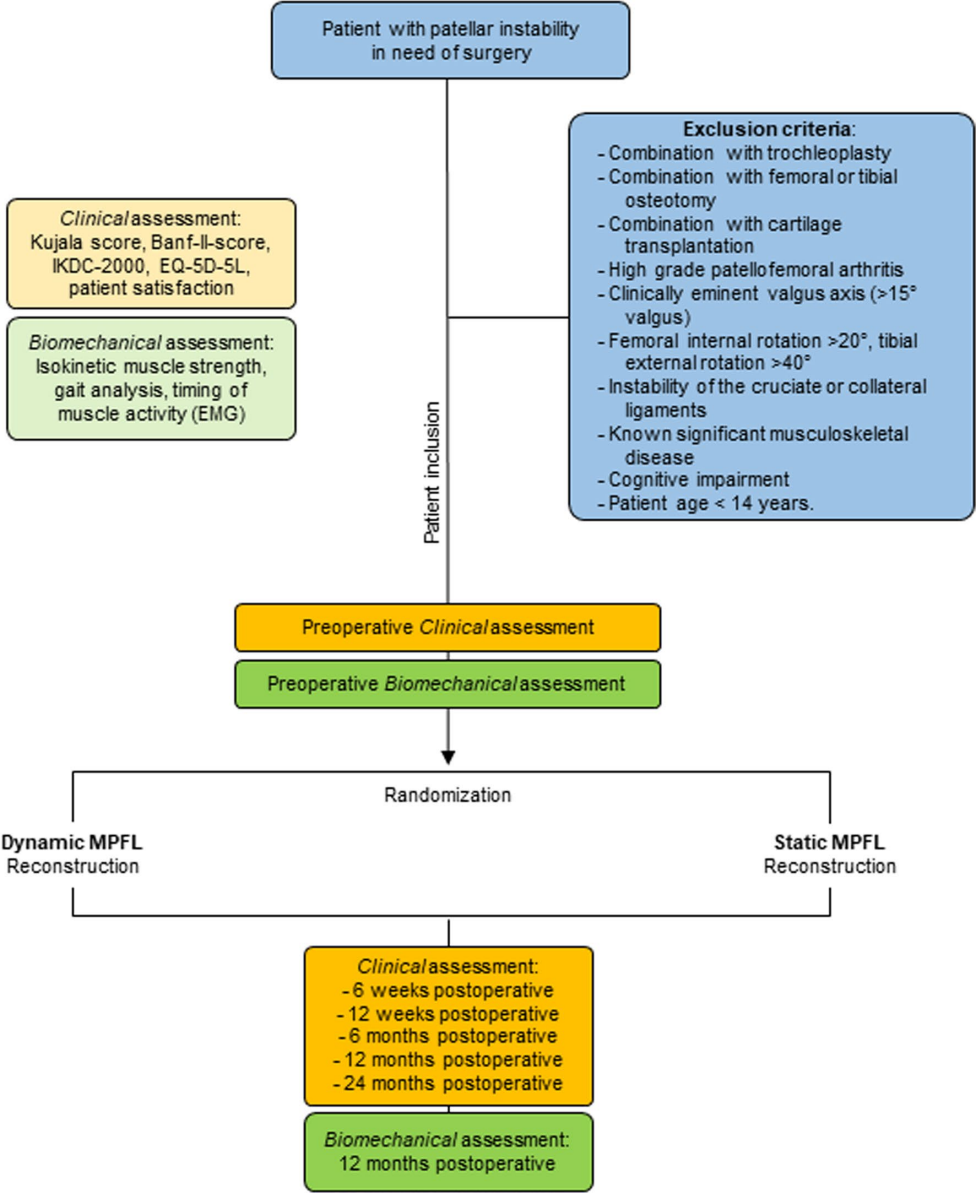
Study procedures to investigate clinical and biomechanical outcome after dynamic and static MPFL reconstruction

Data of the patients will be collected pre- and postoperatively, and all patients will be in contact with the study staff for 2 years (Fig. 2). All patients fulfilling the inclusion criteria will be enrolled consecutively, thus avoiding selection bias. Participants will be followed up for 2 years with a total of five follow-ups at 6 weeks, 12 weeks, 6 months, 12 months and 24 months. Preoperative magnetic resonance imaging (MRI), upright radiographs, surgical reports and patient files will be evaluated. Patients who fulfil the inclusion criteria but refuse participation will be documented in a separate screening log.

### Dynamic and static MPFL reconstruction technique

We will compare the established static MPFL reconstruction technique according to Schöttle et al. [20] with the dynamic reconstruction technique according to Becher et al. [29]. Both techniques use the gracilis tendon for patella stabilization. The static Schöttle-technique harvests the autologous gracilis tendon and inserts the armed tendon at the medial femur condyle (slightly anterior to an elongation of the posterior femoral cortex in between the proximal origin of the medial condyle and the most posterior point of Blumensaat’s line) and at two insertion points at the medial margin of the patella (superomedial corner of the patella and the midpoint of the medial margin of the patella). A fixation is performed with three suture anchors. In the dynamic Becher-technique the distal gracilis tendon is detached periosteal at its anatomical insertion. The freed tendon end is armed and redirected between the quadriceps fascia and the joint capsule to the patella, using the sartorius fascia as a hypomochlion. The fixation of the tendon at the mid third of the medial margin of the patella is performed with a suture anchor according to Bartsch et al. [45].

### Measurements

All study related data will be entered into and stored using the REDCap web-based electronic data capture system [46]. The digital collection system Heartbeat will be used to administer the patient reported outcome questionnaires and data will be transferred to REDCap.

#### Clinical assessment

*Clinical outcome* Assessments will be performed in a preoperative screening, at the hospitalization time and at five postoperative follow-ups. Patient reported knee function, anterior knee pain and quality of life will be recorded by the Kujala-score [35], Banff-II-score [36], IKDC-2000 [36] and the EQ-5D-5L [37]. Moreover, pain level using the numeric rating scale, operation and hospitalization time and general satisfaction with treatment outcome will be assessed.

*Surgical outcome* Surgical outcome will be assessed as: (1) recurrent patella dislocations: the number and time of dislocations will be recorded based on patient recall; (2) revision surgery: number, type and time interval from the MPFL surgery to revision surgery will be recorded based on our operation schedule or patients recall; and (3) other occurring complications (e.g., infection, wound healing disorder): number, type and time interval from MPFL surgery to occurring complication will be recorded based on patients recall and clinicians examination.

#### Biomechanical assessment

Biomechanical outcome will be assessed preoperatively and 1 year postoperatively by assessing isokinetic muscle strength, gait asymmetry, single-legged drop landing, and timing of muscle activity. First, participants will warm up by walking for 5 min at self-selected speed on a treadmill.

Muscle strength will be measured bilaterally using a dynamometer (Biodex System 4 Pro: Biodex Medical Systems, Shirley, NY, USA). For the knee, maximum isokinetic flexion and extension torques will be collected between full extension and 90° flexion at a movement speed of 60°/s (5 repetitions) and 240°/s (15 repetitions) [47]. Maximum joint torques in each movement direction will be recorded for each joint and normalized to body weight. During these tests, electromyographic (EMG) data will be collected using a 16 channel EMG system (myon AG, Schwarzenberg, Switzerland, sampling rate 2400 Hz). Surface electrodes will be placed bilaterally on the vastus medialis and lateralis, semitendinosus, biceps femoris, gracilis, gluteus medius, tibialis anterior, and gastrocnemius medialis muscles following the guidelines of the SENIAM project (Surface Electromyography for the Non-Invasive Assessment of Muscles) [48] and Lovell et al. [49].

Participants will then perform an instrumented gait analysis on an overground walkway with two embedded force plates (Kistler force plate 9260AA6, Kistler AG, Winterthur, Switzerland; sampling rate 2400 Hz) and on a treadmill with an embedded plantar pressure plate (h/p/cosmos, Zebris FDM-T, Isny, Germany; 7168 sensors; area, 1.5 * 0.5 m; range, 1–120 N/cm^2^; precision, 1–120 N/cm2 ± 5%; sampling rate, 120 Hz). Simultaneously with the plantar pressure or force data, kinematic and electromyographic (EMG) data will be collected using a 10 camera Vicon system (Vicon, Oxford, UK; frame rate 240 Hz) and the 16 channel EMG system (see above). To assess 3D joint angles, reflective markers will be placed on predefined anatomical landmarks on the pelvis and lower legs [50]. Subjects will complete overground walking trials on the walkway with embedded force plates. Participants will then walk for 2 min on the treadmill at 0% slope at their preferred walking speed and at 1.2 m/s. Additionally, uphill and downhill treadmill walking (positive and negative slope of 10%) will be measured at 85% of the preferred walking speed. For all conditions, kinematic, EMG and pressure data will be recorded. Subsequently, the treadmill speed will be increased to preferred running speed, and data for 2 min running will be recorded.

To assess muscle activity and leg axis stability, a single-leg vertical drop landing will be performed. As a pretest and to get accustomed with the task, patients will first perform a bipedal drop landing. Participants will then be given a verbal description of the single-leg landing task prior to testing. Standing erect upon only the tested leg with the foot in neutral position, participants will step off a 20 cm high platform. Participants will be instructed to land in the center of the force-plate on the tested leg only. To control for countermovement, participants will be restricted to perform the drop landing with hands upon hips and the contra-lateral knee joint flexed to 90°. All more demanding functional task (running, drop landing) will be performed if patients feel comfortable and do not experience pain during these tasks.

Maxima and minima will be calculated for continuous kinematic and kinetic data [51]. EMG data will be filtered with a modified band pass of 15 Hz to 450Hz [52]. For each muscle, the root mean square of the EMG signal for the stance phase of walking and running will be calculated and normalized to the maximum signal intensity obtained during maximum voluntary contraction [53]. Timing of the gracilis muscle will be assessed as on- and offset relative to the gait cycle. According to Stokes et al. [54], gracilis onset will be determined as the time when the processed EMG signal exceeded a threshold of three standard deviations above a baseline mean and as offset, when the processed EMG signal falls below a threshold of three standard deviations above a baseline mean. For all muscle strength, gait spatio-temporal, and muscle activity parameters, the asymmetry index (AI) will be calculated according to Hodt-Billington et al. [55] as

$$AI= \left|1- \frac{Limb_{lower\_value}}{Limb_{higher\_value}}\right|$$

For all kinematic and kinetic parameters (measured in degrees respective Nm/kg), the asymmetry index will be defined as

$$AI= \left|\mathrm{Limb_{higher\_value}}-\mathrm{Limb_{lower\_value}}\right|$$

#### Statistical analysis

The study involves a series of quantitative outcomes and different types of hypotheses. Hypotheses about differences between patients treated with dynamic or static technique will be addressed by describing and visualizing the distribution of the corresponding variables in each patient group and by quantifying the group differences by the difference in mean values with 95% confidence intervals and p-values. Hypotheses concerning the association between two variables will be addressed by visualizing the joint distribution of variables in scatter plots and quantifying the association by partial correlations coefficients with 95% confidence intervals. Hypotheses concerning a change over time will be addressed by visualizing the distribution of individual differences and quantifying the change over time by the mean difference with 95% confidence intervals and p-values. Hypotheses concerning the association in change over time between two variables will be addressed by visualizing the joint distribution of differences in scatter plots and quantifying the association by correlations coefficients with 95% confidence intervals. In addition, the relation to baseline values will be examined. Patient characteristics will be tabulated for each patient group.

#### Sample size calculation

We based our sample size calculation on the primary outcome, the Kujala score. To date, no high-quality study (randomized trial) on the difference in change in Kujala score between dynamic and static MPFL reconstruction is available. However, in a prospective randomized control trial, Kang et al. [56] compared two different tensioning techniques for static MPFL reconstruction. They reported an effect size of 7.8 for the improvement in the Kujala score for both groups and a group difference in change in Kujala score of 1.9. Based on biomechanical considerations, we expect a larger improvement in patients stabilized using the dynamic MPFL reconstruction. Assuming a 3.8-point improvement in the Kujala score in patients with dynamic MPFL reconstruction compared to patients with static MPFL reconstruction, 54 patients (27 per group) are required to detect a significant difference in change in Kujala score between the two groups with 80% power at a significance level of 5%. We expect a dropout rate of about 10%, and will thus we will target a study cohort of 60 patients (30 per group).

## Discussion

The proposed study will provide the first biomechanical data on the muscle transfer procedure for dynamic MPFL reconstruction and compare these with the established static reconstruction technique. The results of this study can be considered as a first randomized trial evaluating the effectiveness of both techniques and providing evidence for surgery decision-making. Pre- and postoperative biomechanical evaluations will be performed to assess muscle strength and lower extremity kinematics, kinetics and timing and intensity of muscle activity during gait. Most biomechanical studies on the patellofemoral joint focus on describing the static patellar position and joint reaction force by using methods such as computer tomography [57], MRI [58] or cadaveric studies [59] without acknowledging dynamic muscle driven knee motion. This is important to consider in the muscle transfer procedure of dynamic MPFL reconstruction: we anticipate that the gracilis muscle activity will adapt to its new role as patella stabilizer. The capability and efficacy of muscle transfer procedures have been described for other procedures such as rectus femoris transfer in children with cerebral palsy [39, 40]. The gracilis muscle reaches forces up to 300 N from 0° to 60° knee flexion y^41^ indicating that the gracilis muscle is adequate for patella stabilization. With this study, we will provide much needed information on knee biomechanics after dynamic versus static MPFL reconstruction to provide evidence to support orthopedic surgeons in evidence-based decision-making in their quest for surgical techniques most favorable for their patients.

## Data Availability

Not applicable.

## Abbreviations

MFPL: Medial patellofemoral ligament
MRI: Magnetic resonance imaging
AGA: Society for Arthroscopy and Joint-Surgery
PIS: Patella instability severity score
